# Reconstruction of metabolic pathways by combining probabilistic graphical model-based and knowledge-based methods

**DOI:** 10.1186/1753-6561-8-S6-S5

**Published:** 2014-10-13

**Authors:** Qi Qi, Jilong Li, Jianlin Cheng

**Affiliations:** 1 Department of Computer Science, University of Missouri, Columbia, MO 65201, USA; 2 Informatics Institute, University of Missouri, Columbia, MO 65201, USA

## Abstract

Automatic reconstruction of metabolic pathways for an organism from genomics and transcriptomics data has been a challenging and important problem in bioinformatics. Traditionally, known reference pathways can be mapped into an organism-specific ones based on its genome annotation and protein homology. However, this simple knowledge-based mapping method might produce incomplete pathways and generally cannot predict unknown new relations and reactions. In contrast, ab initio metabolic network construction methods can predict novel reactions and interactions, but its accuracy tends to be low leading to a lot of false positives.

Here we combine existing pathway knowledge and a new ab initio Bayesian probabilistic graphical model together in a novel fashion to improve automatic reconstruction of metabolic networks. Specifically, we built a knowledge database containing known, individual gene / protein interactions and metabolic reactions extracted from existing reference pathways. Known reactions and interactions were then used as constraints for Bayesian network learning methods to predict metabolic pathways. Using individual reactions and interactions extracted from different pathways of many organisms to guide pathway construction is new and improves both the coverage and accuracy of metabolic pathway construction. We applied this probabilistic knowledge-based approach to construct the metabolic networks from yeast gene expression data and compared its results with 62 known metabolic networks in the KEGG database. The experiment showed that the method improved the coverage of metabolic network construction over the traditional reference pathway mapping method and was more accurate than pure ab initio methods.

## Introduction

A metabolic pathway is a network of related chemical reactions catalyzed by enzymes that collaboratively produce or degrade one or a few metabolites. Reconstruction of metabolic networks (pathways) plays an important role in studying biological systems. Together with other types of biological networks, metabolic pathways can help decipher relationships between genotype and phenotype, and elucidate essential mechanisms underlying cellular physiology [1].

Most known metabolic pathways stored in the pathway databases such as the Kyoto Encyclopedia of Genes and Genomes (KEGG) [2,3] have been manually curated from the literature. The high-quality manual annotations of metabolic pathways are valuable resources for studying metabolisms, but they only account for a small portion of pathways in most organisms. Therefore, automatic computational reconstruction of metabolic pathways has been an important problem to solve in bioinformatics and computational biology. And a number of methods have been developed to address the problem [4-8].

The most common approach for metabolic pathway construction is based on mapping a group of gene and protein sequences of an organism to known reference pathways [9-11] according to sequence homology. The matched reference metabolic pathways serve as templates to position the genes and proteins (e.g. enzymes) in order to construct the metabolic pathways that they participate in. This approach often can predict several highly likeable reactions in a partially mapped pathway, while leaving substantial unfilled gaps in the pathway because no gene and proteins can be mapped to unfilled regions [12,13]. The other shortcoming of the approach is that it generally cannot predict new reactions that do not exist in a reference pathway.

Ab initio methods that do not use known reference pathways, on the other hand, aim at inferring metabolic pathways from gene expression data or other data sources. Most of these methods employ probabilistic inference methods such as graphical models and Bayesian networks [14-20] in one way or another. The ab initio methods can draw insights directly from biological condition-dependent data sets to construct metabolic pathways, thus have the capability to predict new relations and reactions. However, these methods tend to predict a lot of false positives, resulting in very noisy predictions. The problem is particularly severe when they are applied to construct large metabolic pathways.

Another kind of ab initio methods represents a metabolic network with ordinary differential equa-tions (ODEs) that capture the dynamics of chemical concentrations in a metabolic system. The ODEs can include feedback loops that are often difficult for other methods to handle. Koza et al. reported a method using genetic programming to construct ODEs [21]. A recent study [22] applied the symbolic regression to automatically generate ODEs. The ODE-based methods tend to be computationally intensive, thus might not scale easily up to large metabolic pathways.

In this work, we combine the strengths of both knowledge-based methods and probabilistic graphical model-based methods together in a novel fashion to improve construction of metabolic pathways. The protein-protein relationships underlying metabolic pathway networks are inferred by probabilistic inference methods under the constraints of knowledge extracted from existing reference pathways in the KEGG database. The knowledge includes individual gene products relationships and chemical reactions observed in the KEGG database, which are used as restraints to control the pathway network construction process. That is, instead of exploring any possible relationship between any two genes / proteins in consideration, the Bayesian networks learning method only sample from the observed relationships during the probabilistic pathway construction process. This knowledge-constrained inference not only drastically reduces search space, but also improves prediction accuracy. Since almost all the reactions in a pathway likely exist in one of many other pathways, the knowledge-constrained sampling generally does not compromise the coverage of pathway construction. And because individual relationships and reactions drawn from all other pathways rather than only ones in a reference pathway are considered in construction, the method is able to predict new relations and reactions not existing in a reference pathway. This new way of using known metabolic pathway information sets our method apart from traditional metabolic pathway mapping methods. Our experiment on constructing the metabolic pathways for yeast from scratch showed the performance of the method compared favorably to or was complementary with the pathway mapping methods.

The rest of the paper is organized as follows. The Method section presents the method. The Results section describes and discusses the experiments and results. The Conclusion section summarizes the work and proposes some future directions.

## Methods

**Figure 1:**
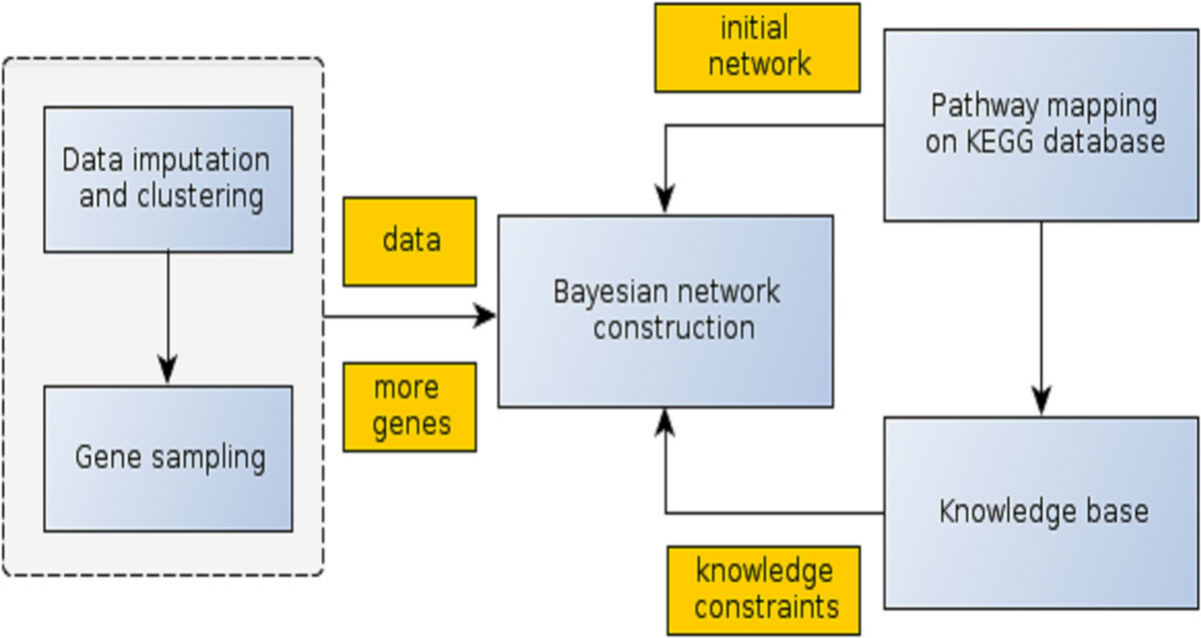
**The main components of the metabolic pathway construction method and their relationships**. The central component is the Bayesian network construction method. Starting from an initial metabolic pathway generated by mapping expressed genes to the KEGG pathway database, it stochastically resamples metabolic networks by drawing reactions and relations based on gene expression data and the reaction/relation knowledge base. The reaction/relation knowledge base is comprised of individual reactions and relations present in any pathway in a pathway database like KEGG regardless of which pathway or species they come from. By using all the observed reactions and relations as knowledge restraints to guide sampling, the method can more effectively and efficiently reconstruct metabolic pathways in three aspects: (1) refining an initial pathway that is largely complete; (2) reconstructing a large portion of an initial pathway when it is largely incomplete; and (3) constructing a completely de novo pathway when the initial pathway has very few or no reactions / relations.

Our method integrates gene expression data of a species, its gene product (protein) sequences, and known metabolic pathways in the KEGG database to construct metabolic pathways. Figure 1 shows main components used by the method and their relationships. A metabolic network is constructed by learning a Bayesian probabilistic network from gene expression data, initial pathway mappings, and all the observed individual relations and reactions in the KEGG database.

Generally, given gene expression data and gene product sequences (i.e. protein sequences) of an organism, the method first maps genes into reference pathways to construct initial seed pathways, and then the seed pathways will be extended and refined by sampling more genes from gene groups clustered according to gene expression data. If the seed pathway contains only a few or no reactions / relations, the method can reconstruct a completely new pathway. The pathway construction is carried out by Bayesian network learning methods under constraints of existing gene-gene relations extracted from all the related pathways in the KEGG database. The details of the metabolic construction method are described in the sub sections below.

### Representation and construction of pathways

Our metabolic network construction method has two main steps: (1) mapping genes to the reference pathways to construct initial seed pathways; and (2) extending and refining initial pathways based on gene expression data and observed relations and reactions. The first step, which is similar to other sequence-homology based methods, obtains initial pathway networks by mapping the genes against KEGG's reference pathways.

The KEGG database [2,3] has a list of metabolic pathways manually curated for many organisms. Met-abolic pathways in KEGG are comprised of enzymes and related chemical reactions, where enzymes denoted by Enzyme Commission (EC) numbers are linked with KEGG Orthology (KO) numbers. The KO numbers are a uniform system to represent ortholog groups, which appear as nodes on visualized metabolic pathway maps. Genes of an organism can be mapped into KO numbers through the gene / protein sequence mapping web service provided by KEGG. With an assignment of genes to KO numbers, the genes can be matched with reference pathways in KEGG in order to construct initial organism-specific pathway maps. Specifically, it marks up those nodes on the reference pathways that contain any of the KO num-bers assigned to the genes. Because the initial mapping usually only recovers a small part of pathways, our method uses the initially mapped pathways as starting points for constructing more complete or even new pathways by adding more genes and relations through the Bayesian network structure learning in the second step.

The second step is to expand the initial pathways by adding more relevant genes, relations and reactions. In order to sample genes that are more likely to participate in the same pathway, genes are first grouped into clusters according to the similarity between their expression data. We used an Expectation-Maximization clustering method implemented in Weka [23] to cluster genes. The method is based on a Gaussian mixture model and determines the number of clusters by cross validation. The gene cluster that contains more genes in an initial mapped pathway than other clusters will be selected as a pivotal cluster for the pathway. The probability of selecting genes from the pivotal cluster is the highest. And the probability of sampling a gene from other clusters is inversely proportional to their distances to the pivot, i.e., the shorter the distance is, more likely that genes in the cluster are selected. And the number of genes to sample is determined by the average number of genes in the reference pathways.

Given all the genes in the current networks including newly sampled ones, a set of knowledge constraints related to all the genes are extracted from the KEGG database. The knowledge set consists of two sub sets. One set includes all possible relationships between the genes that should be included into predicted pathways, and the other sub set contains those which new relationships and reactions can be sampled probabilistically from. Any other relationships not included of the two sub sets will be absolutely excluded. The first subset of relations can be obtained from the initial mapped network structures. The second subset can be derived from all the observed relations in the KEGG database. It contains all existing relationships in the KEGG database between ortholog groups (represented by the KO numbers) of the selected genes. The details about constructing the relation knowledgebase for all the genes of a species are described in the next sub-section. The KO relations can be transformed into gene relations through the mapping from genes to KO numbers. Given a gene set, a collection of all the existing relationships among them can be retrieved from the knowledge base.

The two sets of extracted knowledge constraints are provided as another input besides gene expression data for Bayesian network learning methods for pathway predictions. The purpose is to use the constraints to restrain the search space of reactions and relations of Bayesian probabilistic sampling. Therefore, on one hand, our constrained probabilistic pathway construction is different from typical ab intio sampling techniques that explores the unconstrained pathway space composed of all the pairwise reactions and relations, which is often time consuming and more error-prone. On the other hand, our method samples from all observed relations and reactions between orthologs of genes extracted from whole database rather than the ones extracted from only one reference pathway as traditional pathway mapping methods do. The relation-level knowledge sharing across all the pathways of all the organisms is new and enables our method to take advantage all the existing knowledge of pathways to predict new relations and reactions.

Given the constrained sampling space for a group of genes, we use Bayesian Networks (BNs), a classic type of probabilistic graphical models, to construct metabolic pathways. Reactions and relations (e.g. enzymatic relation) between proteins in metabolic pathways are represented by directed edges in a Bayesian network / graph (G). Given the gene expression data (D), the objective is to find such a graph that achieves the highest score in the following equation:

$$Score\left(G:D\right)=l\left({\hat{\theta }}_{G}:D\right)-\frac{\text{log}M}{2}Dim\left[G\right]$$

This score is also known as the Bayesian Information Criterion. The ${\hat{\theta }}_{G}$ is the maximum likelihood estimator for the model given G and D. The $l\left({\hat{\theta }}_{G}:D\right)$ is log likelihood of data given the G. M is the data size (e.g., number of gene expression experiments). $Dim\left[G\right]$ represents the dimension of G, which equals to the number of independent parameters in G, i.e. total number of parameters used to define the Gaussian distribution of each node representing a gene's expression value conditioned on its parent nodes. More technical details can be found in the book by [24]. The underlying assumption is that the expression value of a gene obeys the linear Gaussian probability distribution given the expression values of its parent genes in G.

The graph search space is explored by manipulating its structure through three types of local operations on edges: adding, removing, and reversing. The search strategy is based on a greedy algorithm. In each iteration, it explores all possible local operations on the current graph, and chooses the move of yielding the highest-score to generate a new graph structure. The process will continue until the score cannot be further improved.

The knowledge-based sampling approach aims to restrain the graphical structure exploration within a small bound space out of the overall search space. The edges appearing in an initial mapped reference network would be kept during the process of graph structure exploration, and a local operation will not generate an edge that does not exist in the knowledgebase.

#### Constructing relation knowledgebase

The purpose of building such a knowledgebase is to generate a realistic set of relations and reactions for pathway predictions, which restricts the relation search space only to observed relations in order to drastically improve search quality and efficiency.

The knowledgebase contains a collection of relations and reactions associated with a list of KO numbers. In order to build such a knowledge base for a target organism, we first map its genes to KO numbers in KEGG database, and then extract the relations and reactions associated with the mapped KO numbers from all the KEGG reference pathway maps.

Specifically in this work, we entered all the genes in Saccharomyces Cerevisiae (yeast) into the KEGG automatic annotation server (KAAS) [9] to map the genes to KO numbers. During the KO number mapping process, yeast genes and pathways in the KEGG database were excluded from being mapped to. The KAAS' output included gene-to-KO assignments and a list of pathway maps that contain any of the assigned KO numbers. The pathway maps were presented in the format of the KEGG Markup Language (KGML), which represent pathway networks in terms of graph objects comprised of KO-number-based nodes and associated relations and reactions as edges.

Based on the KO number assignments and the resulted reference pathway maps, a knowledgebase can be constructed by extracting all the relations and reactions pertaining to the KO numbers. Each relation / reaction includes upstream and downstream entry nodes, a relationship type, and possibly related chemical compounds. Those KO-based entry nodes denote enzymes in metabolic pathway networks, and each of them may contain multiple KO numbers. The type information indicates the kinds of relation or reaction, such as protein-protein or enzyme-compound, etc. Generally, one reaction includes a pair of substrate and product.

### Results and discussion

The objective of the experiment was to evaluate the performance of the knowledge-based probabilistic inference approach for metabolic pathway construction by comparing its predicted yeast metabolic pathway networks with real yeast metabolic pathways existing in KEGG pathway database. The new approach was compared with the standard homology-based mapping approach. The yeast gene expression data set used in this experiment was downloaded from [25].

#### Experiment design

**Figure 2:**
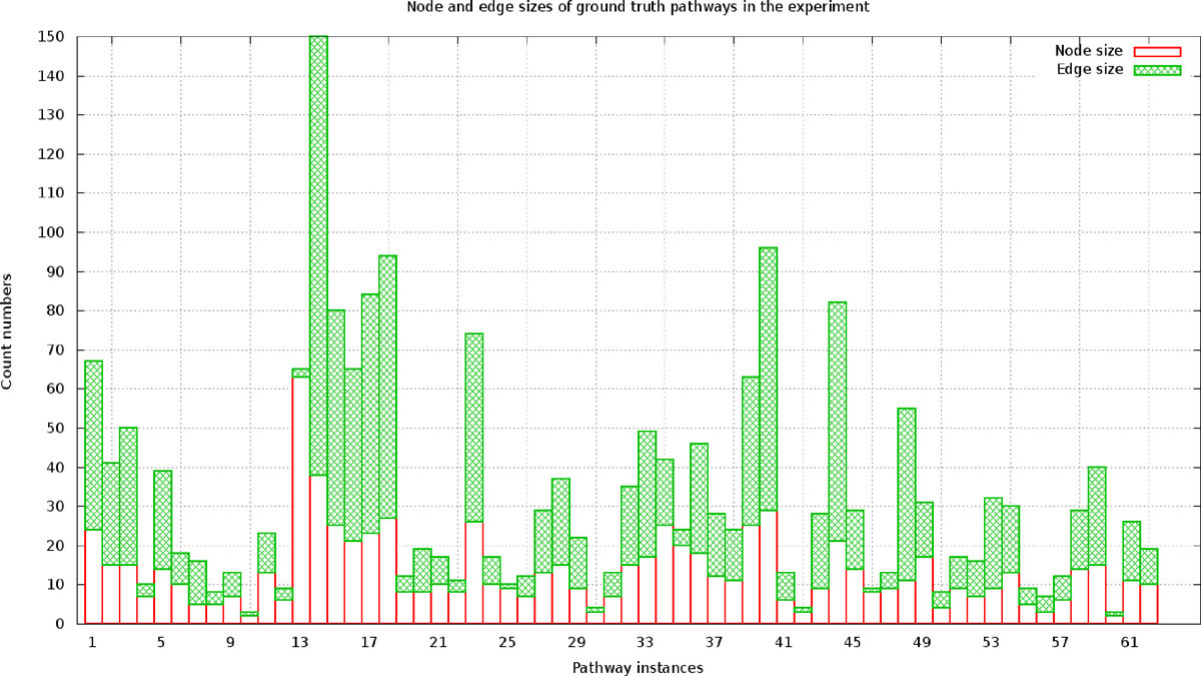
**Statistics for the targeted yeast pathways used in experiments**. X-axis denotes the indices of the pathways ranging from 1 to 52. Y-axis denotes the number of nodes or edges in each pathway.

In order to evaluate the pathways predicted by the computation methods, we collected a list of known yeast metabolic pathways in the KEGG database as presumably true pathways. Totally 68 pathways were found, six of which were removed from the experiment because the nodes in their graphical pathway maps were not connected at all. The 62 remaining pathways served as target (or "true") pathways in the experiment. The statistics about the numbers of the node and edge in the target pathways were reported in Figure 2.

In order to generate input information for predicting the yeast metabolic pathways, the procedure de-scribed in the Method Section was applied to obtain gene-to-KO assignments and construct a relation knowledgebase for the yeast genes. The gene expression data set was preprocessed to filter out those genes with more than 20% missing values. The imputation of missing values for the rest of the data set was carried out with a R program by [26]. The resulted data was classified into 34 clusters by an EM-based method implemented in Weka [23]. The gene clusters were used to guide the gene sampling.

The yeast genes were mapped onto the reference metabolic pathways through the gene-KO assignments. The reference pathways share the same graphical structures with their corresponding specific yeast metabolic pathways, except that their nodes were represented in KO numbers. These initially mapped network structures comprised of the mapped genes and their relations were used as start points by the Bayesian network learning-based methods to construct more complete pathways. Besides the genes appearing in the initially mapped pathway network, another set of genes was sampled from the gene clusters. These two sets of genes were merged together to form a pool of candidate genes to predict metabolic pathway networks.

The knowledge constraints about relationships of the candidate genes in the relation knowledgebase were provided for the Bayesian network structure learning method to select relations between the genes. Given the input genes and the knowledge constraints, we used a Bayesian networks structure learning tool implementing the knowledge-based method [27] to predict metabolic pathways. We applied a score-based heuristic searching method for learning the pathway networks. It searches and scores for networks by altering their graphical structures, and finds the highest scored network (see Section 2.1 for details). The supplied input of knowledge constraints to the learning method was assumed to facilitate the search by restraining it within a realistic exploration space.

#### Results

**Figure 3:**
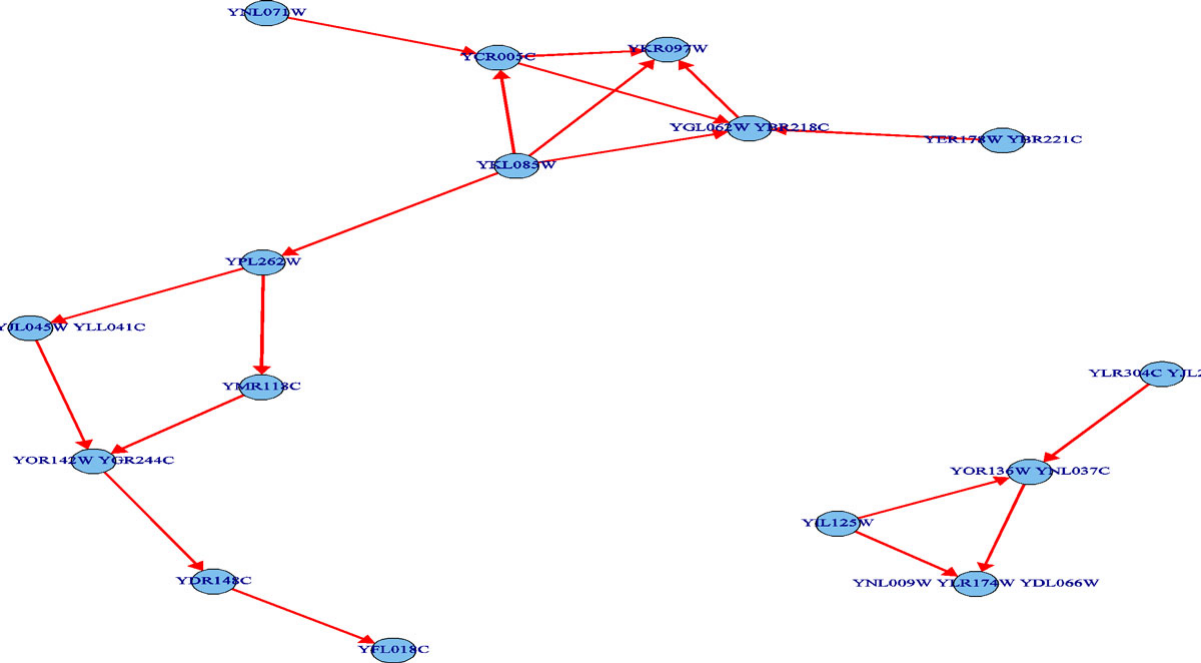
**A target network: the citrate cycle metabolic pathway's underlying relation network in KEGG**.

**Figure 4:**
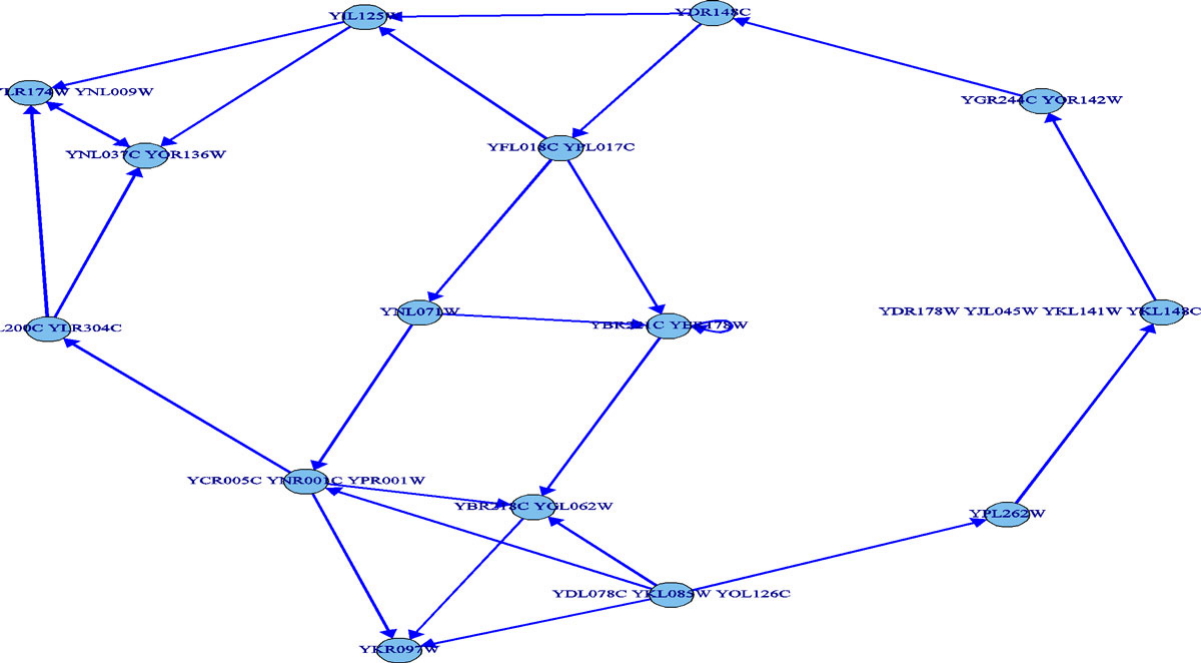
**Resulted network of simple mapping-based method for the targeted citrate cycle metabolic pathway network**.

**Figure 5:**
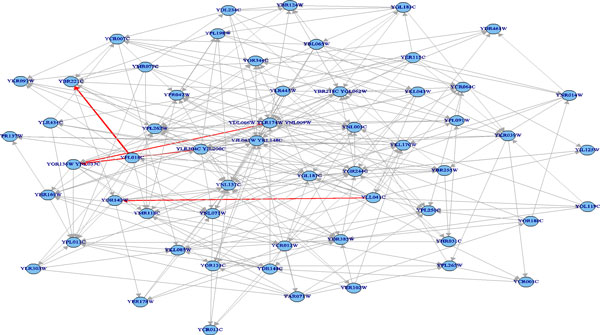
**Resulted network of probabilistic pathway inference without knowledge constraints for the targeted citrate cycle metabolic pathway network**. Red edges denote correct relationships.

**Figure 6:**
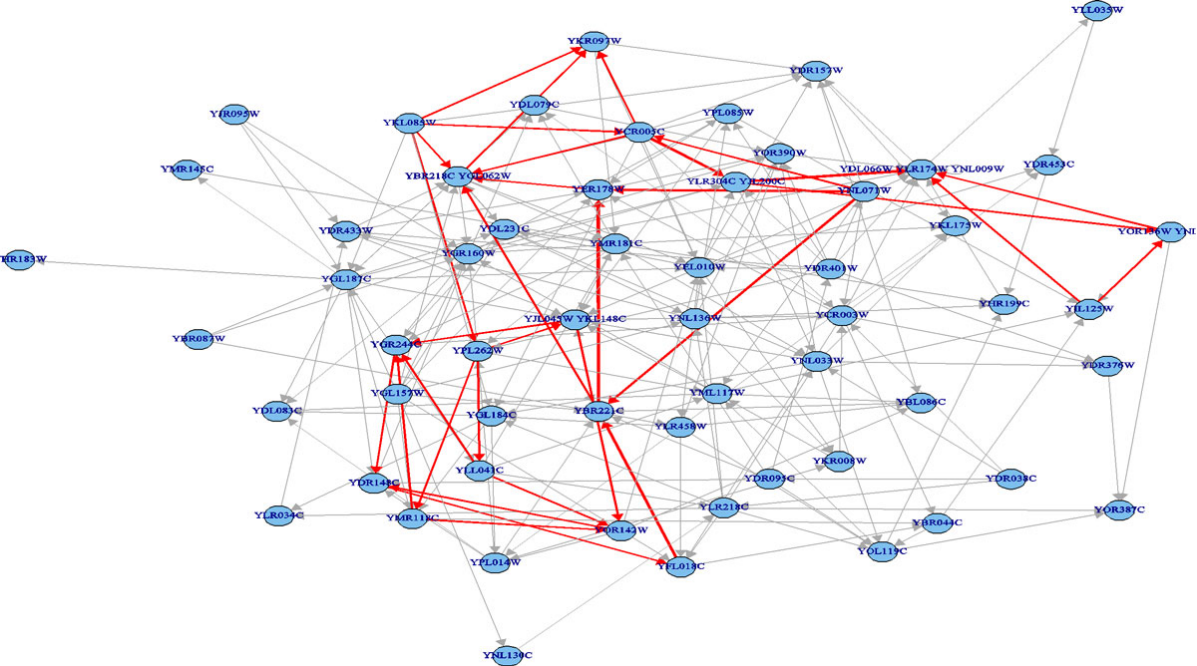
**Resulted network of probabilistic pathway inference with knowledge constraints for the targeted citrate cycle metabolic pathway network**. Red edges denote correct relationships.

**Figure 7:**
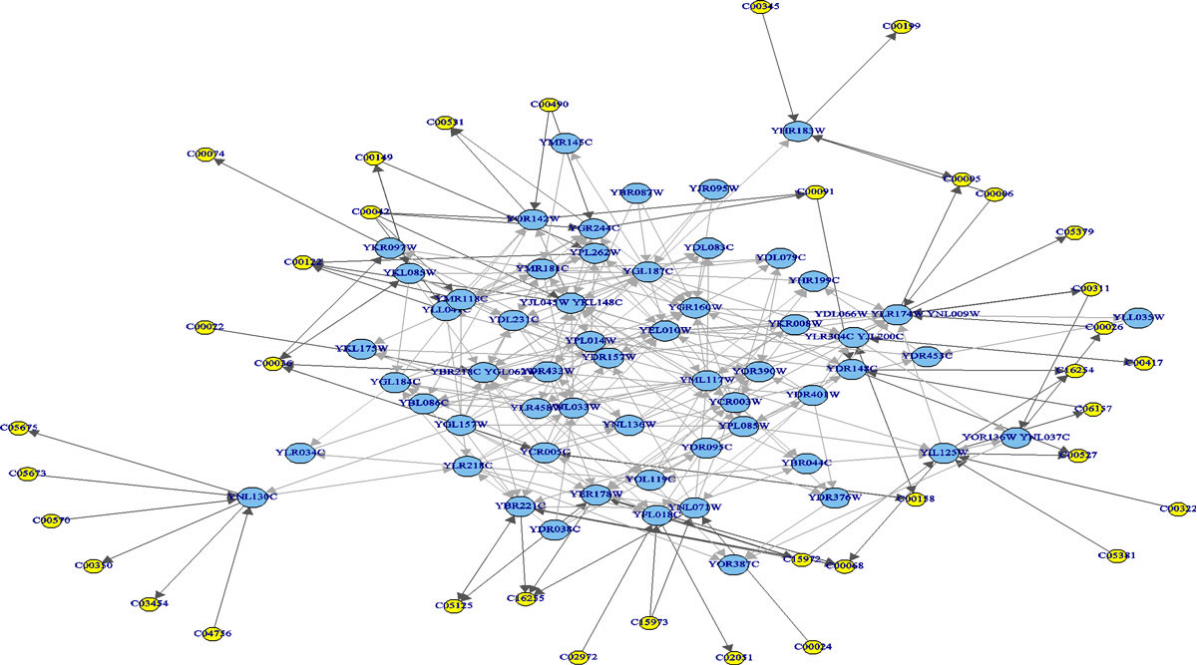
**Resulted metabolic pathway after attaching chemical compounds on to the predictive pathway network in Figure 6**.

The predicted pathway networks were comprised of directly connected nodes (genes), which represent the network structures of gene-product relationships underlying the metabolic pathways. The predicted results were compared with the 62 target yeast metabolic pathways obtained in the KEGG database. The comparison was conducted on relational edges presented both in target and predicted metabolic network structures. For example, given the underlying relation network of KEGG's Citrate cycle metabolism as target network shown in Figure 3. Figure 4, Figure 5 and 6, respectively, show the resulted networks from the mapping-based method, the probabilistic inference method without knowledge constraints, and the one with knowledge constraints. The red-marked edges highlight the correct relations existing in a target network. Figure 4 shows that the simple mapping method recovered a portion of correct relations. Without knowledge constraints, the predicted network in Figure 5 has only a few correctly predicted edges and a large number of falsely predicted ones. The probabilistic inference method with knowledge constraints predicts more correct relation edges than the other two methods (see Figure 6). The predicted relation network was then processed to generate the metabolic network as shown in Figure 7, by adding on relevant chemical compounds from the knowledgebase.

**Figure 8:**
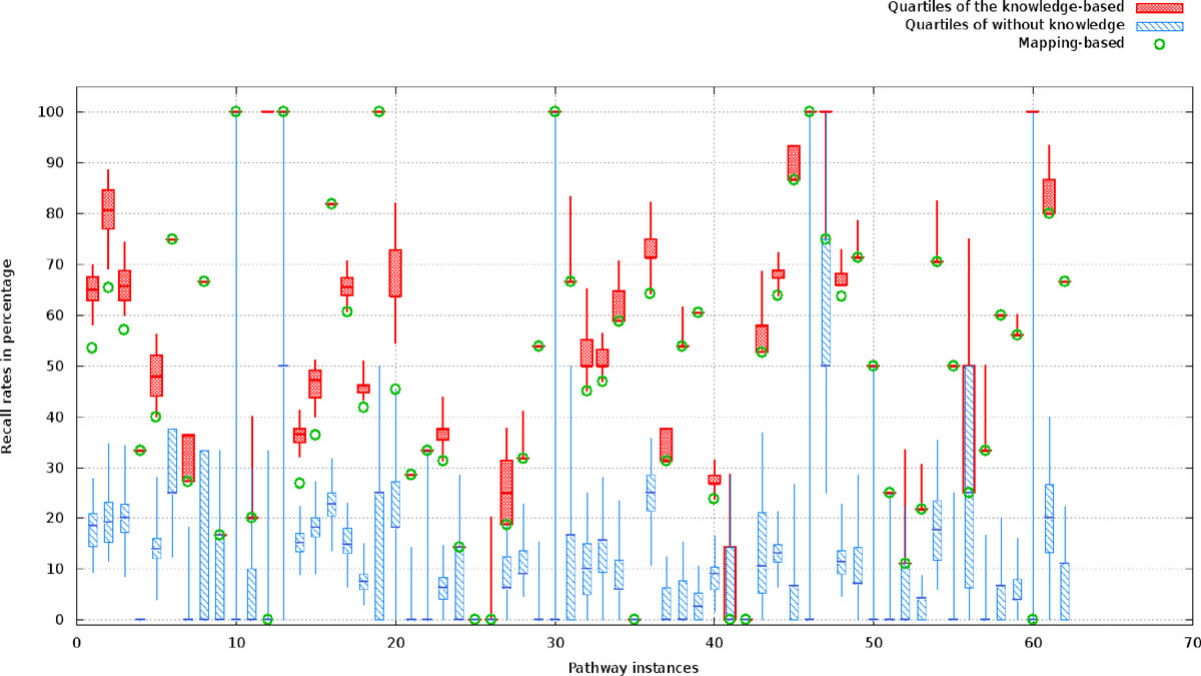
**The recall (the percentage of edges in a ground-truth graph recovered by its counterpart predicted graph) distributions for predictions of three methods: probabilistic sampling with knowledge constraints, probabilistic sampling without knowledge constraints, and the simple mapping-based method without sampling**. One hundred runs were carried out for each of the pathway instances.

**Figure 9:**
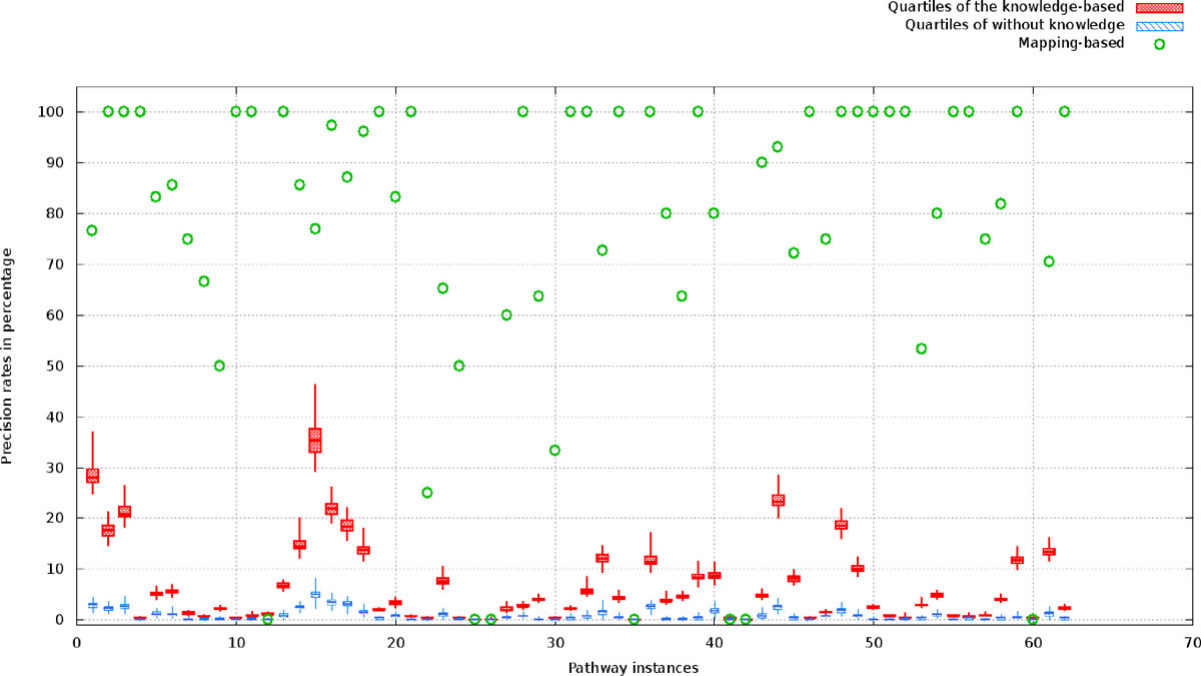
**The precision (the percentage of edges in a predicted graph that exist in its ground-truth graph) distribu-tions for the mapping-based method and the two predictive methods: probabilistic sampling with knowledge constraints, probabilistic sampling without knowledge constraints**. One hundred runs were carried out for each of the pathway instances.

A batch of the same experiments was carried out on the 62 target yeast metabolic pathway networks in KEGG. Figure 8 shows the recalls (i.e. the percent of relations in a target pathway corrected predicted) of the three methods: probabilistic pathway inference with knowledge constraints, probabilistic pathway inference without knowledge constraints, and the simple mapping-based method without inference. Figure 9 reports the precision (i.e. the percent of predicted relations that were correct) of these methods. Since the mapping-based method uses all of the genes to map against nodes on a reference pathway, it does not predict any new edges, but simply takes edges between mapped nodes. Thus, the mapping method tends to have higher precision at the expense of recall. Each of the two probabilistic inference-based methods was run 100 times with randomly sampled genes, resulting 100 predictions for each pathway. Based on the predictions, the minimum, maximum, median, quartile at 25 percentile, and quartile at 75 percentile were calculated and drawn in Figure 8 and 9.

The results in Figure 8 demonstrate that the probabilistic inference method with knowledge constraints generally predicts more correct relations than the simple mapping-based method, which generally performs better than the inference method without knowledge constraints. The knowledge-based probabilistic inference predictions outperformed the mapped-based method in more than 60% of testing instances in terms of recall, while achieving basically the equal recall in the remaining 40% cases. The results indicate that the knowledge-based probabilistic inference can effectively expand the initial mapped networks by adding more correct relations 60% times. In Figure 9, we compared the prediction precisions of the probabilistic inference methods with or without knowledge constraints and the mapping-based method. It shows that incorporating knowledge constraints into the network inference consistently improves the precision of predictions over that without knowledge constraints. Since the mapping-based method did not predict any new relations rather than mapped ones, its precision is higher than the probabilistic network inference methods, which serves as the potential upper bound of predicting new relations beyond the homology mapping methods.

### Conclusions and future work

In this work, we developed a new method combining the probabilistic Bayesian inference with observed relations and reactions to improve the completeness and accuracy of metabolic network construction. The results of this study indicate that the knowledge-based network inference approach was effective in predicting yeast metabolic pathways. It can extend an initially mapped reference pathway by selectively incorporating new relations / reactions in the relation knowledgebase extracted from all the pathway data in the KEGG database, which partially overcomes one major bottleneck of the most widely used pathway mapping methods. Furthermore, the knowledge-based network inference method consistently performs better than the pure ab initio probabilistic network inference method without exploiting the knowledge constraints in terms of both recall and precision, suggesting constraining the ab intio search space using relations / reactions extracted from other pathways is an effective way to improve the de novo prediction of a metabolic pathway. Overall, our experiment demonstrates that combining relation-level knowledge restraints and probabilistic graphical models-based inference is a promising approach to computationally reconstructing of metabolic networks.

It is worth noting that our current implementation of the method did not predict compounds involved in reactions because no compound information was provided. However, the generic compound information can be added into predicted networks by extracting the compounds associated with predicted enzymes from the relation knowledgebase. Furthermore, the information about the compounds known to participate in a metabolic pathway (e.g., metabolomics data), if available, can also be plugged in by comparing them with compounds normally associated with the predicted reactions.

Our method can be extended in different ways. One aspect is to use different methods, such as function similarity rather than the gene expression clustering method to sample genes. Another interesting direction is to investigate the consistency between gene product relationships in predicted pathways and the statistical cause-effect relationships suggested by the Bayesian network inference. Moreover, the Bayesian inference methods used for learning metabolic network structures can be improved in terms of both recall and precision. Furthermore, more relevant data sources, such as protein-protein interaction in the protein interaction databases [28-30], proteomics data, metabolomics data, and protein post-modification data can be integrated with the Bayesian network learning methods to improve the accuracy of metabolic network construction.

In addition to reconstructing metabolic pathways, the novel way of exploiting existing knowledge at the relation level rather than the pathway level can be readily applied to construct other types of biological networks such as signal transduction pathways.

### Competing interests

The authors declare that they have no competing interests.

## Declarations

The publication charges for this article were funded by the University of Missouri, Columbia.

This article has been published as part of *BMC Proceedings *Volume 8 Supplement 6, 2014: Proceedings of the Great Lakes Bioinformatics Conference 2014. The full contents of the supplement are available online at http://www.biomedcentral.com/bmcproc/supplements/8/S6.
